# Effect of *Lonicera caerulea* var. *emphyllocalyx* Fruit on Biofilm Formed by *Porphyromonas gingivalis*

**DOI:** 10.1155/2019/3547858

**Published:** 2019-12-10

**Authors:** Masaaki Minami, Hiroshi Takase, Mineo Nakamura, Toshiaki Makino

**Affiliations:** ^1^Department of Bacteriology, Graduate School of Medical Sciences, Nagoya City University, 1 Kawasumi, Mizuho-ku, Nagoya, Japan; ^2^Core Laboratory, Graduate School of Medical Sciences, Nagoya City University, 1 Kawasumi, Mizuho-ku, Nagoya, Japan; ^3^Nakamura Pharmacy, 7-North5-1 Nango-Dori, Shiraishi-ku, Sapporo, Japan; ^4^Department of Pharmacognosy, Graduate School of Pharmaceutical Sciences, Nagoya City University, 3-1 Tanabe-dori, Mizuho-ku, Nagoya, Japan

## Abstract

*Porphyromonas gingivalis* is an important pathogenic anaerobic bacterium that causes aspiration pneumonia. This bacterium frequently forms biofilms in the oral cavity and in respiratory tract-associated medical devices. Bacterial colonization that occurs in association with this biofilm formation is the main reason for incurable aspiration pneumonia. The *Lonicera caerulea* var. *emphyllocalyx* (LCE) fruit has been used in folk medicine in Hokkaido, the northern part of Japan. The aim of this study was to elucidate one of the antimicrobial mechanisms of LCE methanol extract (LCEE)—the inhibitory effect of LCEE on biofilm formation by *P. gingivalis.* Our results show that LCEE significantly reduced biofilm formation by three different *P. gingivalis* isolates in a concentration- and time-dependent manner that were quantified by the adsorption of safranin red. When LCEE was added to biofilms already formed by *P. gingivalis*, LCEE did not degrade the biofilm. However, treatment with LCEE significantly promoted the removal of existing biofilm by vibration compared to that of control. We also confirmed biofilm formation in LCEE-treated *P. gingivalis* in tracheal tubes using scanning electron microscopic (SEM) analysis. Cyanidin 3-O-glucoside (C3G), one of the components of LCE, also inhibited the formation of biofilm by *P. gingivalis* in a concentration-dependent manner. Our results reveal that LCEE may be an effective antibacterial substance for *P. gingivalis*-induced aspiration pneumonia because of its role in the suppression of bacterial biofilm formation in the oral cavity.

## 1. Introduction

Healthcare-acquired infections are a major cause of mortality and morbidity. According to the existing data, about 10% bedridden patients in developed countries contract hospital-acquired infections [1]. Healthcare-acquired pneumonia, especially ventilator-associated pneumonia, is the leading cause of death in intensive care units (ICUs), with a mortality rate from one-fourth and three-fourth [2].

Aspiration pneumonia is a severe lethal condition caused by aspiration of oral bacteria during medical procedures or by misswallowing of food. It can lead to the development of necrotizing pneumonia or lung abscesses, which may require a prolonged course of antibiotics and surgery [3]. Mortality was about 90% if two or more lobes of the lung were involved and 41% if only one lobe was affected in the previous study [4]. The most common microorganisms isolated from aspiration pneumonia are anaerobes found in the oropharynx [5]. As bacteria are also observed in edentulous elderly patients, the coating of the tongue by bacteria is associated with the risk of aspiration pneumonia [6]. The teeth may also be related as a reservoir for bacterial colonization and nosocomial pneumonia. These anaerobes were found by the colonization of dental plaque in hospitalized intensive care and nursing home [7]. Colonization of the oropharynx and stomach by Gram-negative pathogens increases in critically ill patients immediately after hospitalization [8]. Since bacterial colonization in the mouth and pharynx is a threat for bedridden patients in ICU, several strategies have been used to prevent colonization, such as nonabsorbable antibiotics. Nevertheless, prolonged use of prophylactic antibiotics can lead to increases in resistant organisms and thus is not recommended [9]. Longer ICU admission and longer duration of connection to ventilators are prominent causes of accumulation of infectious agents. Increased duration of patient connection to the ventilator, which is an infection source, causes transmission of infectious agents from the ventilator to the lung [10].

*Porphyromonas gingivalis* is an anaerobic bacterium that is significantly related to periodontitis and several systemic diseases such as aspiration pneumonia. This pneumoniae is responsible for crucial morbidity and mortality in the elderly [11–13]. Numerous clinical case reports and animal models show that this Gram-negative bacterium plays an important role in the development of aspiration pneumonia [14]. Dental plaque biofilm may serve as a persistent reservoir for respiratory diseases. Oral bacteria can be aspirated into the lung to cause aspiration pneumonia. *P. gingivalis* expresses several virulence factors such as lipopolysaccharide (LPS), fimbriae, and cysteine proteinases. In particular, *P. gingivalis* is a popular bacterium isolated from aspiration pneumonia, lung abscesses, and periodontitis in the elderly [15].

*Lonicera caerulea* var. *emphyllocalyx* (LCE), also known as blue honeysuckle or haskap, is a plant belonging to Caprifoliaceae family that grows naturally in cool temperate regions such as high mountains or wet areas in the Northern Hemisphere, such as Hokkaido in Japan, and has been cultivated [16, 17]. Its fruits are purple-colored, hard berries, which are about 1–2 cm long and 1 cm wide and can resist temperatures below −40°C [18]. These fruits of honeysuckle plants have been used in folk medicine in the countries of their origin [19]. Recently, LCE has been widely harvested in many countries including Japan and consumed as a part of the human diet [18]. Previously, we partly described the effect of LCE extract (LCEE) on *Streptococcus pyogenes* infection not only *in vitro* but also *in vivo* [19, 20]. However, the precise mode of antibacterial activity of LCEE against other bacteria has not been unclear.

In this study, we tried to clarify whether LCEE is useful for antiaspiration pneumonia-associated anaerobic bacteria therapy.

## 2. Materials and Methods

### 2.1. Preparation of LCEE

**Figure 1:**
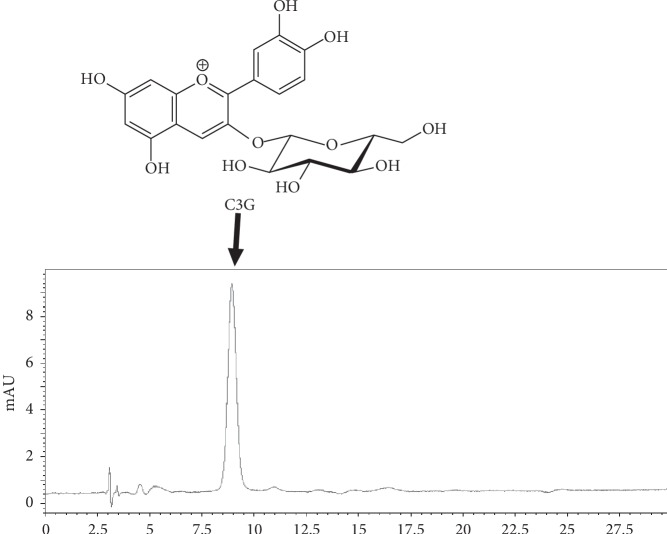
Chromatogram of LCEE. LCEE (5 *μ*g) was injected onto HPLC with the following conditions: system, Shimadzu LC–10A_VP_ (Kyoto, Japan); column, TSK-GEL ODS-80_TS_ (4.6 × 250 mm, Tosoh, Tokyo); mobile phase, 0.5% AcOH/0.5% AcOH in CH_3_OH 85 : 15; flow rate, 1.0 mL/min; column temperature, 40°C; and detection, 520 nm. Peak at 9.0 min was identified as cyanidin 3-O-glucoside (C3G). LCEE: *Lonicera caerulea* var. *emphyllocalyx* extract; HPLC: high-performance liquid chromatography.

*Lonicera caerulea* var. *emphyllocalyx* (LCE) was harvested in Atsuma, Hokkaido, in the northern part of Japan in 2017. The methanol extract of LCE fruit (LCEE) used in this study was the same one as that in our previous study [19, 20]. C3G was purchased from Tokiwa Phytochemical (Sakura, Japan). *Lonicera caerulea* var. *emphyllocalyx* fruit extract (5 *μ*g), cyanidin 3-O-glucoside (C3G, 28.8, 57.5, and 115 ng), was injected to HPLC with the following conditions: system, Shimadzu LC–10A_VP_ (Kyoto, Japan); column, TSK-GEL ODS-80_TS_ (4.6 × 250 mm, Tosoh, Tokyo); mobile phase, 0.5% AcOH/0.5% AcOH in CH_3_OH 85 : 15; flow rate, 1.0 mL/min; column temperature, 40°C; and detection, 520 nm. Retention time of C3G was 9.0 min. The range of C3G was calibrated by the peak area using the least-squares method (*r*^2^ = 0.997) (Figure 1). The concentration of C3G in LCEE was 1.12 (w/w)%.

### 2.2. Bacteria and Chemicals

*Porphyromonas gingivalis* JCM12257 (ATCC33277), JCM8525, and JCM19600 were purchased from RIKEN BioResource Research Center (Ibaraki, Japan). Anaerobes were grown at 37°C under anaerobic conditions (AnaeroPack System, Mitsubishi Gas Chemical, Tokyo, Japan) using Gifu anaerobic medium bouillon (Nissui, Tokyo, Japan), supplemented with 5 *μ*g/mL hemin (Sigma-Aldrich, St. Louis, MO, USA) and 1 *μ*g/mL menadione (Fujifilm Wako Pure Chemical Industries, Osaka, Japan) (GAM) [21]. Ampicillin sodium (Wako Pure Chemical Industries, Osaka, Japan) was used in a final concentration of 50 *μ*g/mL as a positive control.

### 2.3. Bacterial Growth Analysis

Before broth culture analysis, bacteria were incubated in CDC Anaerobe Blood Agar (Nihon BD, Tokyo, Japan) under anaerobic conditions for 48 h. For the determination of the growth inhibitory activity, about 1 × 10^6^ bacteria were incubated in 2 ml of GAM with LCEE or C3G for 24 h. For culturing, 5 mL polypropylene tubes (#34180005D, As-One Corporation., Osaka, Japan) were used. As the determination of bacterial growth, we measured the turbidity of cultured medium (optical density (OD) 600 nm) [21].

### 2.4. Biofilm Assay by Safranin Red Analysis

Biofilm formation was quantified using a polypropylene tube assay specifically for *P. gingivalis* adhesion [22, 23]. Briefly, overnight cultures of *P. gingivalis* were adjusted to 1 × 10^6^ CFU in GAM with or without LCEE and C3G. Aliquots of 200 *μ*L were anaerobically incubated for 72 h at 37°C in a polystyrene tube. To remove planktonic cells, wells were gently washed 3 times with phosphate-buffered saline (PBS, pH 7.2, 0.15 M) and air-dried. After that, remaining bacteria were stained for 15 min with 5 mL of 0.2% (w/v) safranin red. Excess dye was removed by washing 2 times with PBS and then with water. Dye taken up by cells was eluted using 5 mL 95% ethanol, and OD (490 nm) was measured to assess the mass volume of the biofilm. Tubes incubated without bacteria were used as blanks. The absorbance for the blank wells was subtracted from the test values. In dose-dependent analysis, the half inhibitory concentration (IC_50_) was calculated from the least-squares regression line made from 3 points that crossed 50% of the control logarithmic concentration values.

### 2.5. Biofilm Assay by Scanning Electron Microscopic (SEM) Analysis

Scanning electron microscopic preparation was performed as described elsewhere [24]. A tracheal tube (DYND48050J, Medline Japan Inc. Tokyo, Japan) was uniaxially cut at a length of 1 cm, and *P. gingivalis* treated with or without LCEE and C3G were anaerobically incubated in it for 72 h at 37°C. After that, those tubes were immediately placed in 2.5% glutaraldehyde (Nisshin EM, Tokyo, Japan) prepared in 0.1 M phosphate buffer (pH 7.4) for 24 h at 4°C as a prefixation step. They were rinsed 2 times with 0.1 M phosphate buffer (pH 7.4), postfixed using 2% osmium tetroxide (Nisshin EM, Tokyo, Japan) for 2 h at 25°C, and finally rinsed with distilled water. Next, the specimens were dehydrated using graduated concentrations of ethyl alcohol (30%, 50%, 70%, 90%, 95%, and 100%) for 30 min, each followed by absolute alcohol for 30 min. The specimen was dried using the critical point dryer CPD300 (Leica, Wetzlar, Germany). For mounting, carbon conductive paint was used; for specimens, osmium coating was completed using an Osmium Coater (NL-OPC-AJ, Filgen, Nagoya, Japan). Finally, each sample was examined using a microscope (SEM: S-4800) (Hitachi High-Technologies Corporation, Tokyo, Japan).

### 2.6. Statistical Analysis

Experimental data were expressed as mean values with standard deviation (SD). Statistical analysis of the differences between the mean values obtained was performed using unpaired Student's *t*-test for the comparison between two groups or Tukey/Bonferroni's multiple comparison test for differences among multiple groups (EZR version 1.36). The statistical difference was considered significant with *p* < 0.01.

## 3. Results

### 3.1. Bacteria Growth Analysis for LCEE

**Figure 2:**
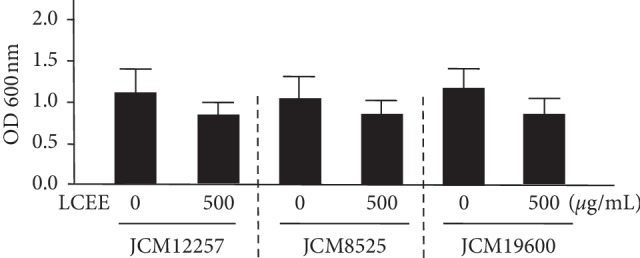
Effect of LCEE on the growth of three *P. gingivalis* isolates. Three *P. gingivalis* isolates (JCM12257, JCM8525, and JCM19600) were treated with or without LCEE (500 *μ*g/mL) for 24 h and their growth was quantified by measuring absorbance at 600 nm. Data represent the mean ± SD (*n* = 3). LCEE: *Lonicera caerulea* var. *emphyllocalyx* extract.

It was evaluated whether LCEE could inhibit the growth of anaerobic bacteria grown in GAM with LCEE. The result showed that LCEE could not inhibit the growth of *P. gingivalis* significantly (Figure 2).

### 3.2. Biofilm Assay with LCEE

**Figure 3:**
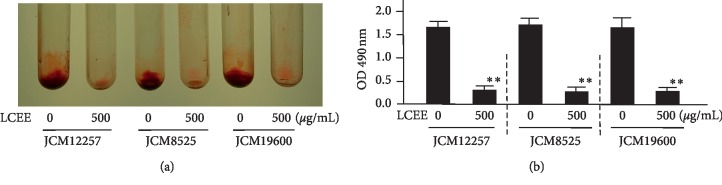
Inhibitory effect of LCEE on biofilm formation by three *P. gingivalis* isolates. Three *P. gingivalis* isolates (JCM12257, JCM8525, and JCM19600) were treated with or without LCEE (500 *μ*g/mL) for 72 h. (a) Image of polypropylene tube. (b) Biofilm formation was quantified by safranin red adsorption at 490 nm. Data represent the mean ± SD (*n* = 3). ^*∗∗*^*p* < 0.01 compared to each untreated group evaluated by Student's *t*-test. LCEE: *Lonicera caerulea* var. *emphyllocalyx* extract.

**Figure 4:**
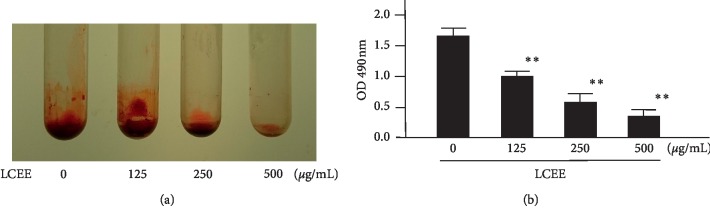
Dose-dependent inhibitory effect of LCEE on biofilm formation by *P. gingivalis. P gingivalis* JCM12257 was treated with or without LCEE (125, 250, and 500 *μ*g/mL) for 72 h. (a) Image of polypropylene tube. (b) Biofilm formation was quantified by safranin red adsorption at 490 nm. Data represent the mean ± SD (*n* = 3). ^*∗∗*^*p* < 0.01 compared to untreated group evaluated by Turkey/Bonnferoni's multiple comparison test. LCEE: *Lonicera caerulea* var. *emphyllocalyx* extract.

**Figure 5:**
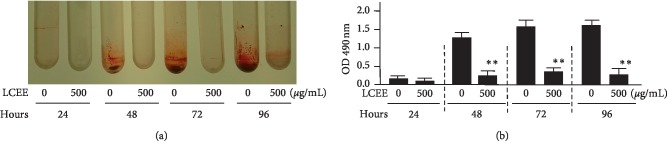
Time-dependent inhibitory effect of LCEE on biofilm formation by *P. gingivalis. P gingivalis* JCM12257 was treated with or without LCEE (500 *μ*g/mL) for 24, 48, 72, or 96 h. (a) Image of polypropylene tube. (b) Biofilm formation was quantified by safranin red adsorption at 490 nm. Data represent the mean ± SD (*n* = 3). ^*∗∗*^*p* < 0.01 compared to each untreated group evaluated by Student's *t*-test. LCEE: *Lonicera caerulea* var. *emphyllocalyx* extract.

**Figure 6:**
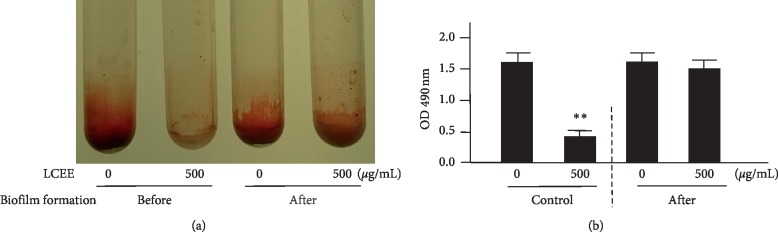
LCEE did not degrade the biofilm already formed by *P. gingivalis*. *P. gingivalis* JCM12257 was treated with or without LCEE (500 *μ*g/mL) for 72 h (control). In order to evaluate the effect of LCEE on the biofilm already formed by bacteria, control bacteria were further treated with or without LCEE for other 72 h (after). (a) Image of polypropylene tube. (b) Biofilm formation was quantified by safranin red adsorption at 490 nm. Data represent the mean ± SD (*n* = 3). ^*∗∗*^*p* < 0.01 compared to each untreated group evaluated by Student's *t*-test. LCEE: *Lonicera caerulea* var. *emphyllocalyx* extract.

**Figure 7:**
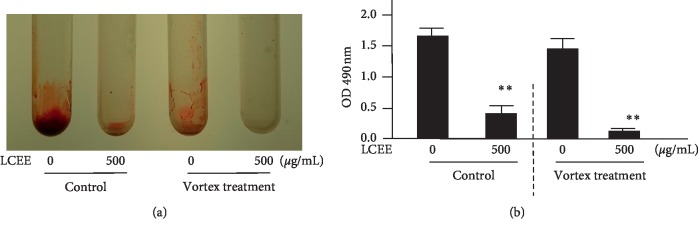
LCEE promoted the degradation of biofilm already formed by *P. gingivalis* subjected to vibrational stimulation. *P. gingivalis* JCM12257 was treated with or without LCEE (500 *μ*g/mL) for 72 h (control). In order to evaluate the effect of LCEE on the biofilm already formed by bacteria, control bacteria were further treated with or without LCEE for other 72 h. Then, a polyethylene tube was vibrated using a vortex mixer for 10 seconds. (a) Image of polypropylene tube. (b) Biofilm activity was quantified by safranin red adsorption at 490 nm. Data represent the mean ± SD (*n* = 3). ^*∗∗*^*p* < 0.01 compared to each untreated group evaluated by Student's *t*-test. LCEE: *Lonicera caerulea* var. *emphyllocalyx* extract.

**Figure 8:**
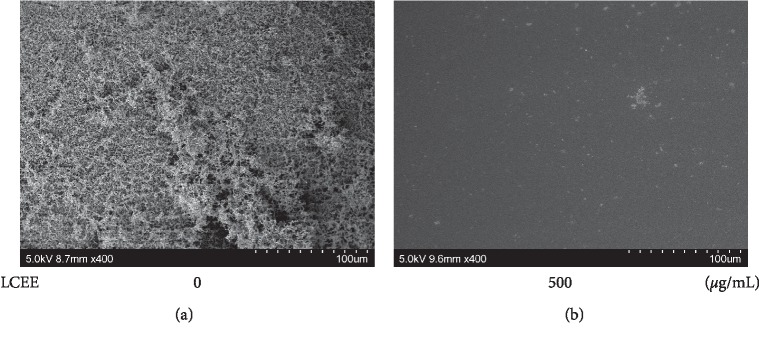
Inhibitory effect of LCEE on biofilm formation by *P. gingivalis* evaluated by SEM assay. *P. gingivalis* JCM12257 was treated with or without LCEE (500 *μ*g/mL) for 72 h. Tracheal tube was involved in culture medium. Biofilm was analyzed by scanning electron microscopy (SEM). (a) Untreated bacteria. (b) LCEE-treated bacteria. LCEE: *Lonicera caerulea* var. *emphyllocalyx* extract.

Although LCEE did not suppress the growth of *P. gingivalis*, we considered other antibacterial effects, including the inhibitory effect on biofilm formation. To evaluate whether LCEE could inhibit biofilm formation or not, pathogenic anaerobes were grown in GAM with LCEE, and the ability to form biofilm in a polypropylene tube was assessed by safranin staining. Safranin red assays showed that untreated anaerobic bacteria formed biofilms intensely. As expected, LCEE significantly inhibited the formation of biofilm by anaerobic bacteria. We confirmed the significant difference of this inhibitory ability of LCEE among 3 bacteria (JCM12257, JCM8525, and JCM19600) (Figure 3). From these universal results, future experiments were focused on *P. gingivalis* JCM12257. More than 125 *μ*g/mL of LCEE inhibited biofilm formation by anaerobic bacteria significantly (*p* < 0.01). The IC_50_ value of LCEE was calculated as 178 *μ*g/mL (Figure 4). We could not find any differences in biofilm formation by *P. gingivalis* by either LCEE-untreated or treated assays at 24 h. However, LCEE inhibited biofilm formation by anaerobic bacteria after 48 h (Figure 5). Thus, it was confirmed that antibiofilm activity of LCEE was present in a concentration- and time-dependent manner. Next, it was examined whether LCEE could affect the biofilm that had already been formed by *P. gingivalis* or not. When *P. gingivalis* was cultured in a medium containing LCEE before biofilm formation, the biofilm of *P. gingivalis* was significantly suppressed. However, the addition of LCEE did not suppress the biofilm already formed (Figure 6). When LCEE was added to the biofilm that had already been formed, LCEE significantly promoted the removal of biofilm by vibration using a vortex mixer for 10 sec compared to control (Figure 7). It was also confirmed the effect of LCEE on biofilm formation in tracheal tubes using SEM scanning. Although *P. gingivalis* formed a monospecies biofilm in control, those cultured with LCEE (500 *μ*g/mL) did not form biofilm significantly (Figure 8).

### 3.3. Bacterial Biofilm Analysis with C3G

**Figure 9:**
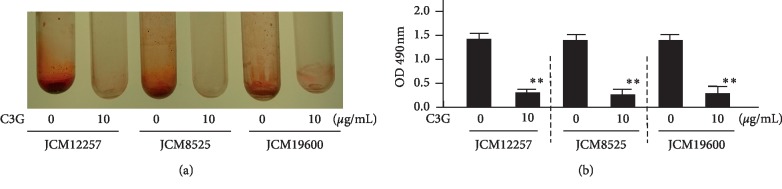
Inhibitory effect of C3G on biofilm formation by three *P. gingivalis* isolates. Three *P. gingivalis* isolates (JCM12257, JCM8525, and JCM19600) were treated with or without C3G (10 *μ*g/mL) for 72 h. (a) Image of polypropylene tube. (b) Biofilm formation was quantified by safranin red adsorption at 490 nm. Data represent the mean ± SD (*n* = 3). ^*∗∗*^*p* < 0.01 compared to each untreated group evaluated by Student's *t*-test. C3G: cyanidin-3-O-glucoside.

**Figure 10:**
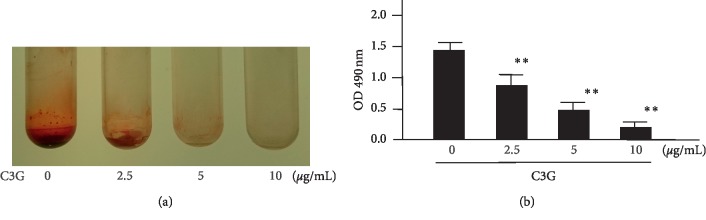
Dose-dependent inhibitory effect of C3G on biofilm formation by *P. gingivalis. P gingivalis* JCM12257 were treated with or without C3G (2.5, 5, and 10 *μ*g/mL) for 72 h. (a) Image of polypropylene tube. (b) Biofilm formation was quantified by safranin red adsorption at 490 nm. Data represent the mean ± SD (*n* = 3). ^*∗∗*^*p* < 0.01 compared to untreated group evaluated by Turkey/Bonnferoni's multiple comparison test. C3G: cyanidin-3-O-glucoside.

**Figure 11:**
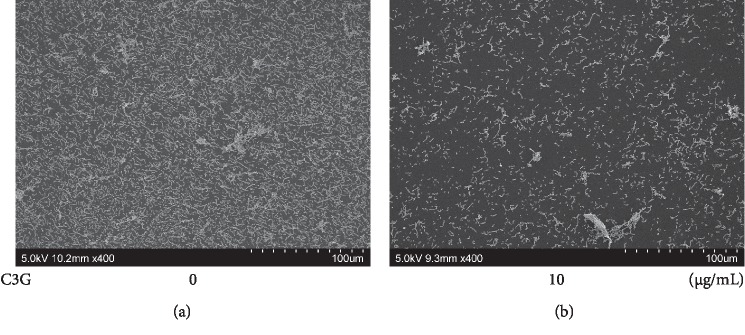
Inhibitory effect of C3G on biofilm formation by *P. gingivalis* evaluated by SEM assay. *P. gingivalis* JCM12257 was treated with or without LCEE (500 *μ*g/mL) for 72 h. Tracheal tube was involved in culture medium. Biofilm was analyzed by scanning electron microscopy (SEM). (a) Untreated bacteria. (b) C3G-treated bacteria. C3G: cyanidin-3-O-glucoside.

Next, it was evaluated whether C3G, one of the components of LCEE, could inhibit biofilm formation by *P. gingivalis* [20, 25]. Three *P. gingivalis* isolates (JCM12257, JCM8525, and JCM19600) were grown in GAM with or without C3G. As expected, C3G exhibited significant inhibitory effects on biofilm formation by three anaerobic bacteria (Figure 9). More than 2.5 *μ*g/mL of C3G significantly (*p* < 0.01) inhibited biofilm formation by anaerobic bacteria in a concentration-dependent manner. The IC_50_ value of C3G was calculated as 3.3 *μ*g/mL (Figure 10). Finally, it was examined the presence or absence of the biofilm inhibitory effect of C3G on bronchial tubes used in clinical practice. SEM scanning showed that although control *P. gingivalis* formed a monospecies biofilm, *P. gingivalis* cultured with C3G (10 *μ*g/mL) did not form biofilm; the difference was statistically significant (Figure 11).

## 4. Discussion

Within the scope of our investigation, this study is the first scientific research on the antibiofilm formation effect of LCEE against *P. gingivalis*. We demonstrated the antibiofilm formation effect of LCEE on polypropylene tubes by safranin red staining and on tracheal tubes by scanning electron microscopy. First, LCEE significantly suppressed the biofilm formation of three independent *P. gingivalis* isolates. Next, our study demonstrated that LCEE significantly inhibited the formation of biofilm by *P. gingivalis* in a concentration- and time-dependent manner. In addition, the pretreatment effect of LCEE was superior to the posttreatment effect. However, we confirmed that applying vibrational stimulation is enough to remove the biofilm even when treated with LCEE after biofilm formation. Furthermore, LCEE reduced the bacterial biofilm in tracheal tubes. The use of C3G, one of the constituents of LCEE, also showed antibiofilm formation effects against three anaerobic bacteria. The effect of C3G was expressed in a dose-dependent manner, and the biofilm inhibitory effect in the bronchial tube was also recognized by SEM analysis. As there are several reports of potential antibiofilm candidate drugs against *P. gingivalis* such as the fruit and seed of *Elettaria cardamomum*, resveratrol, azithromycin, carvacrol and terpinen-4-ol, and curcumin [26–30], there are no established antibiofilm drugs for inhibiting biofilm formation of *P. gingivalis* in western medicine. These results suggest that LCEE treatment would be more beneficial for antibiofilm formation therapy of *P. gingivalis* as well as plaque control.

There are several scientific reports of the antibacterial effect of plant fruits on *P. gingivalis* worldwide. *Phyllanthus emblica* (PE) fruit extract exerts antibacterial effects, and the assessment of *P. gingivalis* revealed significant differences between the PE and control groups [31]. Kapadia et al. showed the antimicrobial activity of banana peel extract on *P. gingivalis*. Using a well diffusion method, *P. gingivalis* showed a 15 mm inhibition zone against an alcoholic extract of banana peel [32]. Seneviratne et al. focused on the mode of antibacterial actions of *Prunus mume* fruit extract against periodontal pathogens. A total of 15 oral pathogens including *P. gingivalis* were investigated to screen the antibacterial activities of *Prunus mume* fruit extract by an agar diffusion assay. *P. gingivalis* was the most susceptible species for *Prunus mume* fruit [33]. Several compounds isolated from the fruits of *Melia toosendan* exhibited significant antibacterial activity against *P. gingivalis* ATCC 33277 [34]. However, we could not confirm the scientific reports of the antibiofilm effect of fruit on *P. gingivalis*. Additionally, our results regarding the antibiofilm effect of LCEE on *P. gingivalis* seem to be beneficial to both basic science and clinical medicine.

We confirmed C3G, one of the compounds found in LCEE, is effective against *P. gingivalis*. Previous reports showed that the content of C3G in LCE fruit was significantly higher than that in other common berries [25]. In the present study, IC_50_ of LCEE was 177 *μ*g/mL. Since LCEE contains 1.12% of C3G, the concentration of C3G in 177 *μ*g/mL of LCEE was calculated to be 1.98 *μ*g/mL. From the approximation formula of the relationship between the antibiofilm activity and the concentration of C3G shown in Figure 10(b), the inhibition percentage of C3G at 1.98 *μ*g/mL was calculated to be 36.3%. Since this concentration was the IC_50_ of LCEE, the contribution of C3G to the activity of LCEE was calculated to be 72.6%. Therefore, C3G plays an important role as the active ingredient in LCEE. To date, there have been few reports of antibacterial effects of C3G against bacteria. C3G suppressed the secretion of CagA and VacA because of intracellular accumulation of CagA and VacA in *H. pylori*. C3G did not inhibit CagA and VacA expression except SecA transcription in *H. pylori*. Although SecA is associated with translocation of bacterial proteins because of the downregulation of SecA expression by C3G, *H. pylori* may reduce the toxin secretion [35]. Yao et al. demonstrated that bayberry fruit extract possessed antibacterial activity against *Salmonella*, *Listeria*, and *Shigella* significantly. The fraction of bayberry with the most activity comprised of flavonoids, which included C3G [36]. Lacombe et al. evaluated the antimicrobial effect of the contents of the American cranberry (the fruit of *Vaccinium macrocarpon*) against *Escherichia coli* O157 : H7; it was demonstrated that anthocyanins produced significant bacterial reductions with minimum inhibitory concentrations of anthocyanins of 14.8 *μ*g/mL (C3G equivalent) [37]. However, it is also clear from our results that C3G has an antibiofilm formation effect, as C3G suppressed bacterial biofilm formation as much as LCE upon SEM analysis. It may be speculated that natural compounds which have antibiofilm formation effects other than C3G are present in LCE. Research on the unknown natural compounds in LCE would be beneficial.

LCEE inhibits the biofilm formation of *P. gingivalis*. Among the components of LCEE, C3G plays an important part in suppressing biofilm formation by this bacterium. Our results reveal that LCEE may be an effective antibacterial substance for *P. gingivalis*-induced aspiration pneumonia because of its ability to suppress bacterial biofilm formation in the oral cavity.

## Data Availability

The data used to support the findings of this study are included within the article.
